# Everybody's Page

**Published:** 1891-09-05

**Authors:** 


### Everybody's Page.

The French officers and men who have visited our coast, have been honoured with a splendid sample of an English summer! During the first days of their visit they will perhaps have thought it pardonable if the Englishman has that disrelish for life with which he is usually credited in their country.

Numerous cases of poisoning from eating ice-creams have been reported lately from the United States. In one instance nearly one hundred people were made very ill at a Church festival. It is a matter for congratulation, that so few children seem to suffer here from the ice-cream vendors who perambulate the streets with their very uninviting looking barrows. But no doubt many more children have their interior economies upset than are ever heard of.

We are apt to grumble at, and be suspicious of the drainage of our cities. The latter attitude is not only a justifiable, but a necessary one if we wish to keep the authorities alive to their duties. But we are apt to forget that the aggregation of great numbers of individuals within restricted areas, and the maintenance of the public health of these masses in relation to drainage and sewage, and the proper condition of cleanliness of the streets, present a very complex and difficult sanitary problem.

Mr. Howard Young, of Hartford, Connecticut, has called attention to the influence which a bedroom with a northern aspect appears to have over him. The facing of his room to the north seems to have a quieting influence on the movement of his heart; sleep is more tranquil and the intellectual faculties much less impressionable. Anyone can, without much difficulty, make this experiment for himself, and if Dr. Young's experience is verified in the generality of cases practitioners will gain some fresh insight into the nervous and organic affections of the heart.

The effects of snake-bite have been combated with success in Australia by the use of strychnine. One of the latest cases of satisfactory recovery is that of a man who had ridden two miles after being bitten, and was in a semi-comatose state when seen. He had taken two ounces of brandy, but his skin was cold and pallid, his pupils were dilated, and he was unable to rise or walk without assistance, and, as is generally the case, had to be kept awake by force. After three injections, within the hour, of a solution of strychnine, his appearance improved, and a profuse perspiration came out. Though the treatment was continued for about ten hours, when after diminished doses at prolonged intervals three-quarters of a grain of strychnine had been administered, the coma returned. During the next six hours another half grain was injected, and the patient recovered.

It appears to be a native custom in South-Eastern Alaska to turn out from her home a woman who is about to become a mother, providing for her only a small, rough shelter made of boards, bark, or canvas as a protection from the weather. Many of the miseries in this world are doubtless caused by human beings themselves, and this is a case in point. In one of these desolate and cold huts, on damp ground, the Alaskan babe is ushered into the world, mother and child unattended either by skilled nurse or physician. According to the *New York Medical Record*, an effort has been made recently by some of the citizens of Sitka to provide better accommodation for the native women, and through their exertions a plan has been matured, and funds have been obtained for the erection of a building in the native village to be known as the St. John's Maternity of Sitka. It is to be hoped that the hospital will be finished before the winter is upon these wretched people.

An American surgeon recommends the use of alcohol as the simplest method for keeping needles from rusting. It is his habit upon buying needles to immerse them in benzine in order to remove grease, then, after running them through a towel, to place them in a wide-mouthed, glass-stoppered bottle filled with absolute alcohol.

The Prussian Minister of Justice has invited the hospital managers of Berlin to consider the cause of the increasing number of suicides in Berlin. Between July 1st and 15th 145 suicides are recorded. The general belief tends to attribute these crimes to indulgence in strong and coarse spirits by the poorer population of Berlin.

The *Therapist* recommends the use of Berberine, irrespective of its corrective properties, as useful for covering successfully the unpleasant and saline flavour of various medicines. It is quite true that many medicines leave a sickly after-taste in the mouth, which occasionally induces boys to the subterfuge of pouring their daily doses out of window instead of down their throats. Doctors, no doubt, are much puzzled sometimes why the drugs they have administered do not have the desired and expected effect. Can this be the reason?

Great dissatisfaction seems to exist amongst medical men in the United States as to the existing coroner system, and a change would probably have taken place some time ago but for the intermixture of politics. It is suggested that the office of Coroner should be abolished and jury service be dispensed with, and that the medical should be separated from the legal duties in all cases involving the examination into the causes of death where crime is suspected. Many of the present difficulties might perhaps be overcome, if the appointment of Coroners were removed entirely from the question of political consideration, and were based only upon their possession of the requisite qualifications.

People are willing and eager, states the *Healthy Home*, to speak in warmest praise of the physician who has brought them safely through some awful crisis of disease; and rightly. But how seldom do they recognise or appreciate that greater skill which detects disease in its early stages, and so promptly and wisely treats it that the patient does not go down into the shadow of death. The greatest skill is habitually displayed in treating the everyday ailments, so commonly regarded as trivial and unimportant; and almost every physician of large experience will tell you that much of his best work passes unrecognised and unthought of. Indeed, his greatest, many times his only reward, comes from the consciousness of a good work well done.

The *Pall Mall Gazette* points out that the present year has for some reason been unusually prolific in deaths under chloroform. Two have been reported during the present week, one at the Middlesex Hospital and the other at Liverpool. The Manchester Infirmary alone has been the scene of four or five similar accidents since the beginning of the year. Juries at coroners' inquests have in all cases returned formal verdicts of death by misadventure, which under the circumstances was all they could do. Nevertheless, the impression is forced upon laymen who have read the medical evidence given at these inquiries that doctors are very much in the dark respecting the action of chloroform upon heart and lungs.

